# Center-specific variation in outcomes for extremely premature, extremely low birth weight neonates

**DOI:** 10.3389/fped.2025.1570542

**Published:** 2025-06-20

**Authors:** Sai Manasa Kalyanam, Jordan R. Kuiper, Theodore J. Iwashyna, Lindsey A. Knake, Esther G. Lee, James L. Wynn, Khyzer B. Aziz

**Affiliations:** ^1^ Department of Pediatrics, Johns Hopkins University, Baltimore, MD, United States; ^2^ Department of Environmental and Occupational Health, George Washington University, Washington, DC, United States; ^3^ Department of Pulmonary and Critical Care Medicine, Johns Hopkins University, Baltimore, MD, United States; ^4^ Department of Pediatrics, University of Iowa, Iowa City, IA, United States; ^5^ Department of Pediatrics, Rush University, Chicago, IL, United States; ^6^ Department of Pediatrics, University of Florida, Gainesville, FL, United States

**Keywords:** neonatal care, extremely premature, extremely low weight, center-specific variation, neonatal practices

## Abstract

The survival rate of neonates born with extremely low birth weight (<1,000 g) and extremely preterm (<29 gestational age) has significantly improved with advances in neonatal care. Despite such advances, outcomes vary widely across neonatal intensive care units due to differences in care practices and patient population. In this study, we examined 1,627 extremely low birth weight and extremely preterm infants admitted to three NICUs across the United States between 2013 and 2023. We evaluated survival and severe intraventricular hemorrhage (SIVH) using statistical models that were adjusted for maternal and neonatal characteristics. Significant differences in outcomes were observed between the centers. These differences were associated with variations in care practices, including resuscitation decisions for the infants. Despite these differences, all centers achieved survival without SIVH for a substantial number of infants, annually. These findings emphasize the need for evidence-based practice-sharing and improvements to ensure better and more consistent care.

## 1 Introduction

There is an increasing number of neonates previously considered “non-viable” due to extreme prematurity and/or low birth weight that survive hospital discharge (1, 2). However, while variation in neonatal center practices is known to be a driver of outcomes, elucidation of how these centers differ is rarely reported. For example, differences between centers may be due to underlying differences in severity of illness among their respective patient populations. However, if differences remain even after accounting for established risk factors as well as the underlying severity of illness, then this may indicate unexplored sources of center differences, including at the practitioner, center, and even state level (e.g., state-specific policies and regulations impacting care). Such information would inform practice, patient counseling, and public policy. The primary objective of this study was to assess center-specific variation in survival and severe intraventricular hemorrhage (SIVH) among extremely low birth weight (ELBW) and extremely preterm (EP) infants across three geographically distinct academic NICUs and to evaluate whether these differences remained significant after adjusting for maternal and neonatal characteristics.

## 2 Methods

We conducted a retrospective cohort study of 1,627 inborn, ELBW (<1,000 g), and extremely preterm (EP, <29 weeks' gestation) infants admitted to three academic NICUs (January 2013–May 2023). All centers were Level IV NICUs that attend and care for similar patients but are located within three geographically and sociodemographically diverse regions of the United States (Midwest, East Coast, and Southeast). This project was reviewed and approved by the institutional review board at all participating institutions. We followed the STROBE guidelines and reporting.

Our primary outcomes were death and severe intraventricular hemorrhage (SIVH, ≥grade 3); the latter is associated with neurodevelopmental impairment (3). We compared descriptive statistics for maternal and neonatal characteristics using the Kruskal–Wallis (continuous) and chi-square (categorical) tests. We used modified Poisson regression models with robust standard error estimation to estimate the unadjusted and adjusted associations of the study center with the risk of death and severe intraventricular hemorrhage (SIVH). Effect estimates were reported as relative risks (i.e., risk ratios) (RRs) and 95% confidence intervals. We included as covariates maternal and neonatal characteristics including maternal age, mode of delivery, antenatal steroid administration, preeclampsia, preterm labor, gestational age (GA), birth weight (BW), birth year, maximum vasoactive-inotropic score (VIS), and 5 min Apgar score (4). Note that the absence and presence of preeclampsia and preterm labor were defined by the International Classification of Diseases (ICD) 9 and ICD 10. We used maximum vasoactive-inotropic score (VIS) during the NICU hospitalization, a metric directly associated with mortality in extremely preterm neonates (5–8), as an objective proxy for quantifying patient-level severity of illness. Additionally, since there is no accepted definition of neonatal hypotension, the administration of vasoactive-inotropic medications also reflects center-specific practices and physician behavior (5). Briefly, VIS was calculated using the same approach as described previously (5), in which all vasoactive-inotropic medication exposures during the observed birth encounter were identified. The resulting VIS was calculated for each hour using the following formula: VIS = dopamine dose (µg/kg/min) + dobutamine dose (µg/kg/min) + 100 × epinephrine dose (µg/kg/min) + 10 × milrinone dose (µg/kg/min) + 10 × vasopressin dose (mU/kg/min) + 100 × norepinephrine dose (µg/kg/min). For the birth encounter, we retained the maximum VIS calculated. One-year mortality follow-up was obtained by querying all encounters for infants born at <24 weeks GA.

We conducted two sensitivity analyses of the main regression models. First, as a sensitivity analysis to the main SIVH regression model, we additionally estimated effect estimates among survivors only, to ensure that any observed associations with SIVH were not driven solely by a higher preponderance of mortality in this group. Additionally, given the large proportion of neonates with scores of 0 for VIS, we ran models using categorical VIS as opposed to continuous. This categorical VIS was operationalized as VIS = 0 (category 1), and then for those with non-zero VIS, we divided observations based on tertiles (tertile 1 = category 2, tertile 2 = category 3, and tertile 3 = category 4). Finally, to visualize time trends for death and SIVH across the three centers, we ran the main regression models but with a quadratic term for birth year, an interaction between birth year and center, and an interaction between the quadratic term and center.

Given that four patients had some missing data for the above covariates, we used multiple imputations by chained equations (*m* = 50 data sets) to impute the missing values for maternal age (*n* = 1), mode of delivery (*n* = 1), and Apgar score at 5 min (*n* = 2). Maternal age and Apgar score were imputed using predictive mean matching in tandem with *k*-nearest neighbors (*k* = 5) while the mode of delivery used logistic regression. All imputation models were informed by the other variables being imputed and the fully observed covariates and outcomes (i.e., death and SIVH), and models were run stratified by center. We ran all final analytic regression models on the 50 imputed datasets and combined effect estimates and 95% CIs using Rubin's rules (9).

## 3 Results

**Figure 1:**
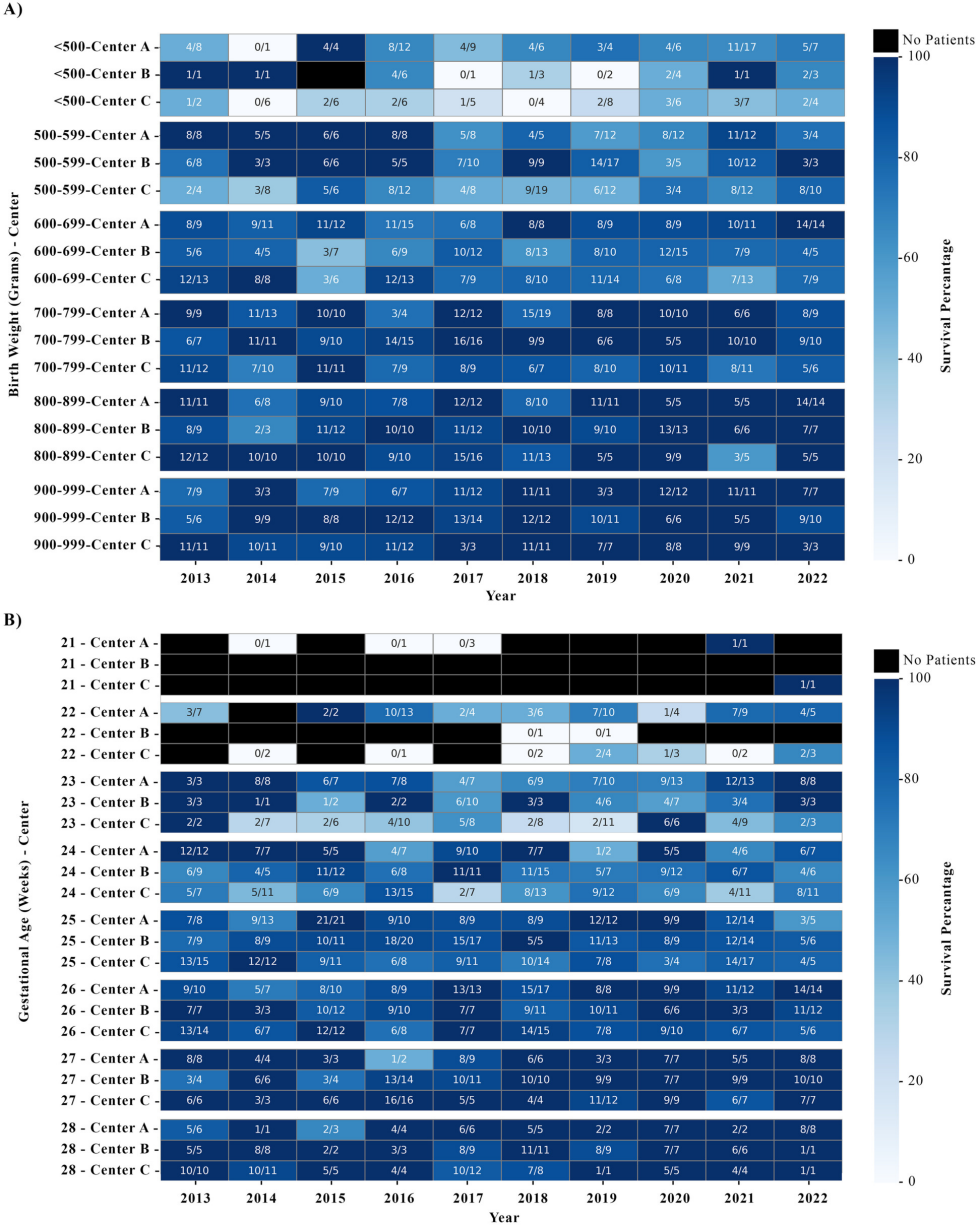
Survival rates categorized by birth weight (<1,000 g, **A**) and gestational age (<29 weeks, **B**) across different centers and years. Each cell contains the number of survivors over the total number of patients. The data presented are for years with information available for all 12 months. Black cells indicate that there were no patients for the specified category.

On average, there were 52.9 ELBW/EP neonates per year for Center A, 46.7 for Center B, and 51.4 for Center C. Using the Kruskal–Wallis test, distributions [median (IQR)] of VIS differed across centers (*p* < 0.001) with values at Center C [15 (8–25)] being higher than Center A [10 (5–20)] and Center B [12 (6.5–20)] (Table 1). Although maternal characteristics of neonates delivered at each of the three centers are statistically significantly different, maternal age was largely comparable between the centers with the overall median (IQR) age among centers being 29 (24–34); however, the incidence of preeclampsia differed significantly across centers with Center A having the lowest incidence (16.5%) and Center C having the highest (60.1%) (Table 1). Similarly for preterm labor, incidence was significantly different across centers with Center A having the lowest occurrence (36.1%) and Center C having the highest (61.0%) (Table 1). It is important to note that the proportions of vaginal deliveries and antenatal steroid administration, despite being statistically significant, were mostly comparable across the three centers (Table 1), although Center A had the highest proportion of vaginal deliveries (34.9%) and antenatal steroid administration (95.5%) while Center B had the lowest for both (25.7% and 90.1%, respectively). Among neonates delivered <23 weeks GA, center-specific standards of care regarding resuscitation led to differences in survival rates at Center B compared with Centers A and C (Figure 1). As shown in Figure 1B, across these three centers, at least 75 ELBW/EP infants survived hospital discharge without SIVH every year.

**Table 1:** Cohort characteristics.

Characteristic	All centers (*N* = 1,627)	Center A (*N* = 582)	Center B (*N* = 514)	Center C (*N* = 531)	*p*-value
Maternal characteristics					
Median age (IQR), years^a^	29 (24–34)	29 (25–34)	31 (26–35)	27 (23–33)	<0.001
Vaginal delivery, no. (%)^b^	503 (30.9%)	203 (34.9%)	132 (25.7%)	168 (31.6%)	0.004
Preeclampsia, no. (%)	553 (34.0%)	96 (16.5%)	138 (26.8%)	319 (60.1%)	<0.001
Preterm labor, no. (%)	784 (48.2%)	210 (36.1%)	250 (48.6%)	324 (61.0%)	<0.001
Antenatal steroids, no. (%)	1,509 (92.7%)	556 (95.5%)	463 (90.1%)	490 (92.3%)	0.002
Neonatal characteristics					
Median BW (IQR), grams	720.1 (591–854)	705 (565–837.5)	750 (620–870)	714.1 (587.4–849.9)	<0.001
Median GA (IQR), weeks	25.6 (24.3–26.9)	25.3 (23.7–26.6)	25.9 (24.7–27.3)	25.6 (24.4–27)	<0.001
Male, no. (%)	781 (48%)	283 (48.6%)	241 (46.9%)	257 (48.4%)	0.827
Median 5 min APGAR score (IQR)^c^	7 (5–8)	7 (6–8)	6 (5–8)	6 (4–7)	<0.001
SIVH, no. (%)	300 (18.4%)	64 (11%)	125 (24.3%)	111 (20.9%)	<0.001
Death, no. (%)	276 (17%)	83 (14.3%)	69 (13.4%)	124 (23.4%)	<0.001
Median length of stay (IQR), days^d^	92 (61–123)	107 (81.2–139)	77 (44–108.2)	89 (60–114.5)	<0.001
Median VIS max (IQR)	12 (6–20)	10 (5–20)	12 (6.5–20)	15 (8–25)	<0.001
Vasoactive-inotropic medication^e^, no. (%)	870 (53.5%)	304 (52.2%)	291 (56.6%)	275 (51.8%)	0.223

BW, birth weight; GA, gestational age; SIVH, severe intraventricular hemorrhage (Grade 3–4); VIS, vasoactive-inotropic score; received vasoactive-inotropic medications.

^a^ Missing data on one patient.

^b^ Missing data on one patient.

^c^ Missing data on two patients.

^d^ Missing data on 50 patients.

^e^ Receipt of vasoactive-inotropic medications.

**Figure 2:**
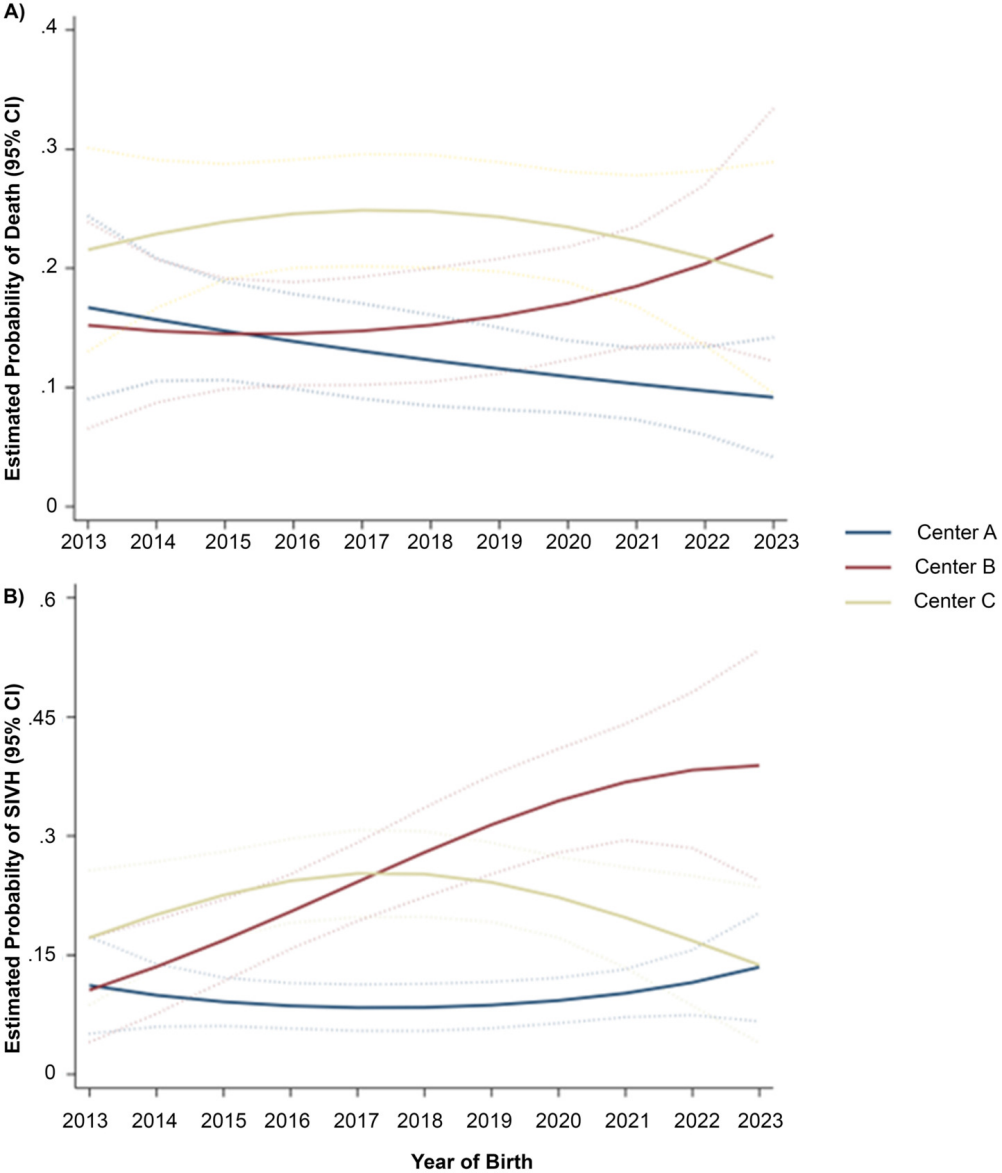
Estimates derived from multiply-imputed, modified Poisson regression model of death **(A)** or SIVH **(B)** that included maternal age, mode of delivery, antenatal steroid receipt, gestational age (GA), birth weight (BW), maximum vasoactive-inotropic score (VIS), preeclampsia, preterm labor, 5 min Apgar score, birth year, and birth year-squared as well as interaction terms between birth year and study center and as well as birth year-squared and study center.

Death occurred in 14.3%, 13.4%, and 23.4% for Centers A, B, and C, respectively (*p* < 0.001), while SIVH occurred in 11.0%, 24.3%, and 20.9% for Centers A, B, and C, respectively (*p* < 0.001). Survival by birth weight (<1,000 g) and gestational age (≤ 28 weeks) varied by center and year (Figure 2). In covariate-adjusted analyses, referent to Center A, neonates had a 35% (RR: 1.35, CI: 0.98, 1.85) or 93% (RR: 1.93, CI: 1.47, 2.52) greater risk of death at Center B or Center C, respectively (Table 2). For SIVH, neonates had a 174% (RR: 2.74, CI: 2.05, 3.67) or 135% (RR: 2.35, CI: 1.74, 3.18) greater risk at Center B or Center C, respectively. In our sensitivity analysis restricted to survivors (*n* = 1,351), the adjusted risk of SIVH among survivors was 247% (RR: 3.47, CI: 2.40, 5.03) or108% (RR: 2.08, CI: 1.36, 3.18) greater at Center B or C, respectively. In our sensitivity analyses using categorical VIS max, effect estimates for center were consistent with respect to the direction of association, although absolute magnitudes were often slightly attenuated (Supplementary Tables S2, S3). The estimated risk of SIVH varied over time across centers, while time trends in risk of death did not substantively differ (Figure 2).

**Table 2:** Unadjusted and adjusted relative risks (95% confidence intervals) of maternal and neonatal covariates and center with death or SIVH (*n* = 1,627).

Variables	Death (Unadjusted)	Death (Adjusted)	SIVH (Unadjusted)	SIVH (Adjusted)
Center B (ref: Center A)	0.94 (0.70, 1.27)	1.35 (0.98, 1.85)	2.21 (1.68, 2.92)	2.74 (2.05, 3.67)
Center C (ref: Center A)	1.64 (1.27, 2.11)	1.93 (1.47, 2.52)	1.90 (1.43, 2.53)	2.35 (1.74, 3.18)
Maternal age (per 1 year)		1.00 (0.99, 1.02)		0.99 (0.97, 1.00)
Cesarean delivery/other non-vaginal delivery (ref: vaginal delivery)		1.28 (1.00, 1.63)		1.14 (0.91, 1.43)
Antenatal steroid administration (ref: no)		0.69 (0.52, 0.91)		1.02 (0.73, 1.42)
Preeclampsia (ref: no)		0.79 (0.63, 0.99)		0.68 (0.52, 0.89)
Preterm labor (ref: no)		1.13 (0.90, 1.43)		1.15 (0.91, 1.45)
GA (per 1 week)		0.83 (0.75, 0.92)		0.73 (0.66, 0.81)
Birth weight (per 500 g)		0.21 (0.13, 0.35)		1.33 (0.84, 2.10)
Apgar score at 5 minutes (per 1 unit)		0.96 (0.92, 1.01)		0.94 (0.90, 0.98)
VIS max (per 1 unit)		1.013 (1.009, 1.018)		1.006 (1.002, 1.01)
Year of birth (per 1 year; centered at 2018)		0.99 (0.95, 1.02)		1.05 (1.02, 1.09)

Models estimated using multiply-imputed, modified Poisson regression.

GA, gestational age; SIVH, severe intraventricular hemorrhage; VIS, vasoactive-inotropic score.

The chart review of infants born at 21, 22, or 23 weeks was feasible at Centers B and C. Of the 30 infants who survived to initial Center B discharge, 2 are known to have died prior to their first birthday, and 26 (87%) returned for some form of healthcare after age 1. Of the 37 infants who survived to initial Center C discharge, none are known to have died prior to their first birthday, and 33 (89%) returned for some form of healthcare after age 1.

## 4 Discussion

These data demonstrate (a) survival without SIVH of at least some infants, even the most premature and smallest birthweights; (b) that the likelihood of such survival varies substantially between peer centers after adjustment for accepted risk factors; (c) and that, among otherwise comparable “peer centers,” standard-of-care decisions regarding resuscitation of infants contributes to observed center-level variation. This finding demonstrates the profound impact policy can have on care delivery and suggests an urgency to develop rigorous evidence-based practice sharing and evaluation, as the degree of heterogeneity between peer centers in practice and outcomes suggests there are opportunities for dramatic improvements in care. Our findings also have implications for tying the basis for reproductive rights to questions of “viability” as they demonstrate that absolute survivability varies across geography and policy, rather than being rooted solely in some aspects of biology.

These results are consistent with previous studies demonstrating center-level variation in outcomes among extremely preterm infants, even after adjustment for neonatal and maternal factors. For example, the NICHD Neonatal Research Network and Vermont Oxford Network have both demonstrated wide variability in survival and morbidity across NICUs, highlighting the influence of local policies and practice patterns (10, 11). Our findings build upon this by incorporating VIS as a means to systematically account for the severity of illness using an objective tool, reinforcing its emerging utility in benchmarking neonatal care. Together, these findings support the need for ongoing collaborative quality improvement and practice standardization across institutions.

We acknowledge limitations, including the possibility that some infants may not have been admitted to the NICU (considered non-viable with or without resuscitation), that there are other adverse outcomes not analyzed here, and that residual confounding may exist for other differences in center-specific referral populations. The magnitude of variation among sites suggests the opportunity for structured, scalable, granular practice-sharing as well as research to improve outcomes in this vulnerable population.

## Data Availability

The datasets presented in this article are not readily available because of the vulnerable patient population. Requests to access the datasets should be directed to kaziz5@jhu.edu.
