# Supplementary material for: Center-specific variation in outcomes for extremely premature, extremely low birth weight neonates
Source: Front Pediatr. 2025 Jun 20;13:1570542. doi: 10.3389/fped.2025.1570542 (PMC12226493; doi:10.3389/fped.2025.1570542)
Supplement: Supplementary file 1 [file Supplementaryfile1.docx]

Supplementary Material

# Supplemental Tables

Supplemental Table 1. Unadjusted and adjusted relative risks (95% confidence intervals) of maternal and neonatal covariates and center with SIVH, among survivors (n = 1351).

| Variable | Unadjusted | Adjusted |
| --- | --- | --- |
| Center B (ref: center A) | 3.15 (2.21, 4.49) | 3.47 (2.40, 5.03) |
| Center C (ref: center A) | 1.66 (1.11, 2.48) | 2.08 (1.36, 3.18) |
| Maternal age (per 1 year) |  | 0.98 (0.96, 1.00) |
| Cesarean delivery/other non-vaginal delivery (ref: vaginal delivery) |  | 1.10 (0.82, 1.47) |
| Antenatal steroid administration (ref: no) |  | 0.93 (0.61, 1.43) |
| Preeclampsia (ref: no) |  | 0.65 (0.44, 0.94) |
| Preterm labor (ref: no) |  | 1.17 (0.87, 1.57) |
| GA (per 1 week) |  | 0.76 (0.67, 0.87) |
| Birth weight (per 500 grams) |  | 1.59 (0.88, 2.89) |
| Apgar score at 5 minutes (per 1 unit) |  | 0.90 (0.85, 0.96) |
| VIS max (per 1 unit) |  | 1.007 (0.999, 1.015) |
| Year of birth (per 1 year; centered at 2018) |  | 1.08 (1.03, 1.14) |

Note: Models estimated using multiply-imputed, modified Poisson regression.

Abbreviations: GA = gestational age; SIVH = severe intraventricular hemorrhage; VIS = vasoactive inotropic score

Supplemental Table 2. Unadjusted and adjusted relative risks (95% confidence intervals) of maternal and neonatal covariates and center with death or SIVH (n = 1627), using categories of maximum VIS^a^.

|  | Death | Death | SIVH | SIVH |
| --- | --- | --- | --- | --- |
| Variable | Unadjusted | Adjusted | Unadjusted | Adjusted |
| Center B (ref: center A) | 0.94 (0.70, 1.27) | 1.06 (0.77, 1.44) | 2.21 (1.68, 2.92) | 2.52 (1.88, 3.38) |
| Center C (ref: center A) | 1.64 (1.27, 2.11) | 1.50 (1.15, 1.96) | 1.90 (1.43, 2.53) | 2.18 (1.61, 2.96) |
| Maternal age (per 1 year) |  | 1.01 (0.99, 1.02) |  | 0.99 (0.97, 1.01) |
| Cesarean delivery/other non-vaginal delivery (ref: vaginal delivery) |  | 1.13 (0.90, 1.42) |  | 1.11 (0.88, 1.39) |
| Antenatal steroid administration (ref: no) |  | 0.71 (0.53, 0.95) |  | 1.02 (0.73, 1.44) |
| Preeclampsia (ref: no) |  | 0.79 (0.64, 0.98) |  | 0.68 (0.52, 0.88) |
| Preterm labor (ref: no) |  | 1.00 (0.80, 1.26) |  | 1.11 (0.88, 1.41) |
| GA (per 1 week) |  | 0.88 (0.80, 0.97) |  | 0.75 (0.68, 0.83) |
| Birth weight (per 500 grams) |  | 0.40 (0.25, 0.64) |  | 1.57 (0.99, 2.47) |
| Apgar score at 5 minutes (per 1 unit) |  | 0.98 (0.93, 1.03) |  | 0.95 (0.90, 0.99) |
| VIS max 2^nd^ category (ref: category 1) |  | 1.83 (1.09, 3.06) |  | 0.93 (0.65, 1.31) |
| VIS max 3^rd^ category (ref: 1^st^ category 1) |  | 3.47 (2.18, 5.52) |  | 1.54 (1.14, 2.07) |
| VIS max 4^th^ category (ref: 1^st^ category 1) |  | 8.85 (5.66, 13.8) |  | 2.00 (1.48, 2.69) |
| Year of birth (per 1 year; centered at 2018) |  | 1.01 (0.97, 1.04) |  | 1.06 (1.02, 1.10) |

Note: Models estimated using multiply-imputed, modified Poisson regression.

Abbreviations: GA = gestational age; SIVH = severe intraventricular hemorrhage; VIS = vasoactive inotropic score

^a^ Categories defined by: VIS max = 0 (category 1), and then tertiles of VIS max among those with non-zero values (categories 2, 3, 4 = tertile 1, 2, 3, respectively).

Supplemental Table 3. Unadjusted and adjusted relative risks (95% confidence intervals) of maternal and neonatal covariates and center with SIVH, among survivors (n = 1351), using categories of VIS max ^a^.

| Variable | Unadjusted | Adjusted |
| --- | --- | --- |
| Center B (ref: center A) | 3.15 (2.21, 4.49) | 3.25 (2.24, 4.73) |
| Center C (ref: center A) | 1.66 (1.11, 2.48) | 1.96 (1.28, 3.00) |
| Maternal age (per 1 year) |  | 0.98 (0.96, 1.01) |
| Cesarean delivery/other non-vaginal delivery (ref: vaginal delivery) |  | 1.09 (0.81, 1.46) |
| Antenatal steroid administration (ref: no) |  | 0.94 (0.61, 1.45) |
| Preeclampsia (ref: no) |  | 0.65 (0.45, 0.94) |
| Preterm labor (ref: no) |  | 1.14 (0.85, 1.54) |
| GA (per 1 week) |  | 0.77 (0.68, 0.88) |
| Birth weight (per 500 grams) |  | 1.79 (0.98, 3.29) |
| Apgar score at 5 minutes (per 1 unit) |  | 0.91 (0.85, 0.96) |
| VIS max 2^nd^ category (ref: category 1) |  | 0.88 (0.59, 1.31) |
| VIS max 3^rd^ category (ref: 1^st^ category 1) |  | 1.38 (0.97, 1.95) |
| VIS max 4^th^ category (ref: 1^st^ category 1) |  | 1.79 (1.19, 2.69) |
| Year of birth (per 1 year; centered at 2018) |  | 1.09 (1.04, 1.14) |

Note: Models estimated using multiply-imputed, modified Poisson regression.

Abbreviations: GA = gestational age; SIVH = severe intraventricular hemorrhage; VIS = vasoactive inotropic score

^a^ Categories defined by: VIS max = 0 (category 1), and then tertiles of VIS max among those with non-zero values (categories 2, 3, 4 = tertile 1, 2, 3, respectively).
